# Multi-Objective Optimization Design of Hybrid Fiber-Reinforced ECC Based on Box–Behnken and NSGA-II

**DOI:** 10.3390/ma18214914

**Published:** 2025-10-27

**Authors:** Xiao Wang, Haowen Jing, Hongkui Chen, Sen Zheng, Fei Yang, Jinggan Shao

**Affiliations:** 1 School of Materials Science and Engineering, North China University of Water Resources and Electric Power, Zhengzhou 450045, China; wangxiao@ncwu.edu.cn (X.W.); jinghaowen11@163.com (H.J.); 13137439050@163.com (S.Z.); 2 Research and Development Center of Transport Industry of Application Technology of High Performance Green Materials, Henan Jiaoyuan Engineering Technology Group Co., Ltd., Zhengzhou 451450, China; chk98@163.com; 3 School of Architectural and Civil Engineering, Zhongyuan University of Technology, Zhengzhou 450007, China; feiyang@zut.edu.cn

**Keywords:** Engineered Cementitious Composites, hybrid fibers, Box–Behnken, NSGA-II, mechanical properties, optimization algorithm

## Abstract

To enhance the effectiveness and precision of design and to produce more low-carbon and high-performance Engineered Cementitious Composites (ECCs), novel hybrid fiber-reinforced high-ductility cementitious composites developed by incorporating a combination of ultra-high-molecular-weight polyethylene fibers (UHMWPE) and basalt fibers (BFs) into the cementitious matrix. Building upon the Box–Behnken design model from Response Surface Methodology (RSM), this study investigates the effects of different water-to-binder ratios and fiber contents on the mechanical properties of hybrid fiber-reinforced ECC. Analysis of variance (ANOVA) was used to validate the regression models. Furthermore, multi-objective optimization of the ECC mix proportion was achieved by employing the NSGA-II algorithm in conjunction with the TOPSIS comprehensive evaluation method. The results indicate that UHMWPE and BFs exhibited a significant positive hybrid effect. The order of factor significance was as follows: The content of ultra-high-molecular-weight polyethylene is greater than that of basalt fiber, and the content of basalt fiber is greater than that of the water–binder ratio. The results of variance analysis show that the regression model has high fitting accuracy. Furthermore, the algorithmic optimization yielded an optimal mix proportion: a water-to-binder ratio of 0.21, UHMWPE fiber content of 1.51%, and BF content of 0.85%. This study provides a valuable reference for the multi-objective optimization design of ECC mix proportions targeting diverse strength and toughness requirements.

## 1. Introduction

Traditional cement concrete is an important building material due to its properties of high strength, good durability, and economy. However, it exhibits limited tensile strength and relatively low failure strain, and it is prone to brittle fracture under tensile, fatigue, and impact loads [1]. In order to improve the toughness of the matrix, many scholars have proposed a variety of strategies and approaches. During this period, based on mesoscopic and fracture mechanics design, Professor Victor Li of the University of Michigan in the United States developed Engineered Cementitious Composites (ECCs), which are a fiber-reinforced cement-based composite material [2]. Its superior ultra-high tensile strain ductility can effectively improve structural performance and enhance structural toughness [3]. Typically, the primary fiber utilized in ECCs is organic polyvinyl alcohol (PVA). In terms of workability, the volume stability of ordinary ECC materials is low, the synergy with other engineering materials is poor, and the production of organic fibers is often accompanied by higher CO_2_ emissions [4,5]. In view of this situation, scholars have developed hybrid fiber-reinforced cement-based composites and carried out research [6].

Hybrid fiber is a direct and effective way to improve the deficiency in single-fiber-reinforced systems and learn from each other. When different types of fibers are incorporated into a cement matrix, as a result of a positive hybrid effect, the advantages of different fiber materials can be brought into play [7]. A thermoplastic polymer, ultra-high-molecular-weight polyethylene (UHMWPE) fiber, offers outstanding mechanical performance. Relative to conventional PVA fibers, UHMWPE fiber has higher effective complementary energy. The PSH of the composite is classified as saturated PSH behavior, a phenomenon that serves to improve the fracture toughness of the cementitious matrix. However, the weak bond strength at the UHMWPE fiber–matrix interface adversely affects crack width control [8]. Basalt fiber (BF) is formed through the melting of basalt ore. The main chemical components are SiO_2_ and Al_2_O_3_. It exhibits notable advantages, particularly its high tensile strength and low production cost combined with strong resistance to chemical corrosion [9]. BF-ECC has excellent compatibility with the matrix, and has a positive effect on crack width control and flexural strength improvement of composite materials [10]. Although the hybrid of these two materials holds the potential for ECC with enhanced strength and strain capacity, research into its specific mix design and performance remains scarce.

Furthermore, the mix design of hybrid fiber-reinforced ECC must comprehensively account for the complex interactions among multiple factors, including the water-to-binder ratio and fiber content. Traditional trial-and-error approaches are often inadequate for efficiently elucidating the underlying mechanisms. Therefore, it is imperative to develop a more effective and precise design methodology. To address the complex interactions inherent in the aforementioned mix design and to establish an effective and precise design methodology, this study was conducted using Response Surface Methodology. Furthermore, a multi-objective optimization of the ECC mix proportion was achieved by employing the NSGA-II algorithm in combination with the TOPSIS comprehensive evaluation method. This approach provides a valuable reference for the design optimization of ECC mixes tailored to diverse strength and toughness requirements.

Response Surface Methodology (RSM) is an optimization method that combines experimental design with mathematical statistics [11]. The Box–Behnken design belongs to the family of experimental designs in the Response Surface Methodology. It is used to find the relationship between an output variable (response) and an input variable (factor), and has good robustness [12]. As an effective data analysis method, Response Surface Methodology has been applied to different experimental designs and data analyses by scholars at home and abroad. Li et al. [13] verified the efficacy of the Response Surface Method, demonstrating that this mathematical modeling optimization technique can effectively reduce the number of experiments while testing interactions between factors. He et al. [14] studied the mix design of recycled aggregate concrete, and used the Box–Behnken design method in the response surface to explore the influence of different cement dosages, recycled aggregate replacement rates, and water consumption rates on the flexural strength and compressive strength of concrete. Enhancement in global search ability is achieved in the non-dominated sorting genetic algorithm (NSGA-II) by introducing the elite retention strategy, and it can generate a uniformly distributed Pareto front solution set, showing excellent convergence performance and robustness. However, the selection of the final solution still has subjective dependence [15]. For multi-objective decision-making problems, the TOPSIS method, grounded in the information entropy principle, is applied to analyze the characteristics of index variations, to construct the objective weight system, and to implement the comprehensive evaluation based on the multi-objective decision-making framework of system engineering; this provides the ability to minimize deviations resulting from subjective weighting [16].

Based on the above, this study used UHMWPE and BF as reinforcing fibers and used the Box–Behnken in the RSM method to design the mix ratio to prepare hybrid fiber high-ductility cement-based composites. This study systematically analyzed how the compressive strength, flexural strength, and equivalent bending toughness of ECC are interactively affected by the water-to-binder ratio, UHMWPE fiber content, and basalt fiber (BF) content. Through the experimental design, numerical fitting, model test, NSGA-II algorithm optimization, and TOPSISI analysis, the comprehensive evaluation of the objective function was completed. The optimal parameter combination solution set of hybrid fiber ECC was obtained and verified with experiments. This is of great significance for cement-based composites to achieve high strength and toughness, as well as satisfy green and sustainable goals.

## 2. Materials and Methods

### 2.1. Testing Material

**Figure 1:**
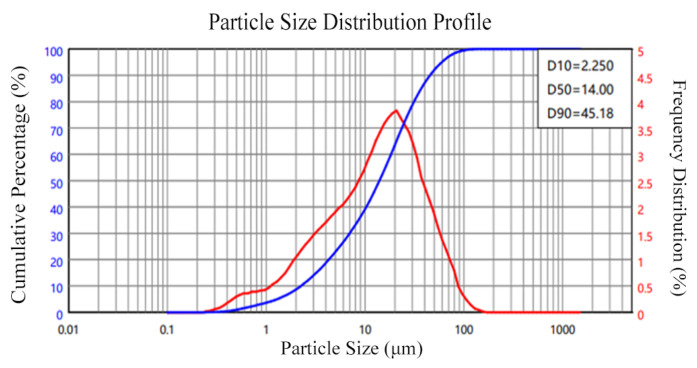
Particle size distribution of cement.

**Figure 2:**
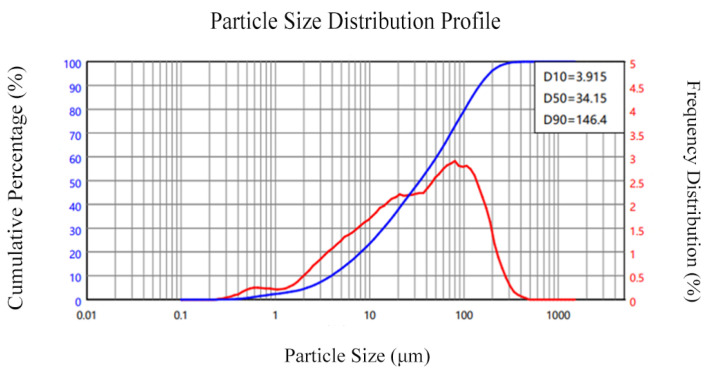
Particle size distribution of fly ash.

**Figure 3:**
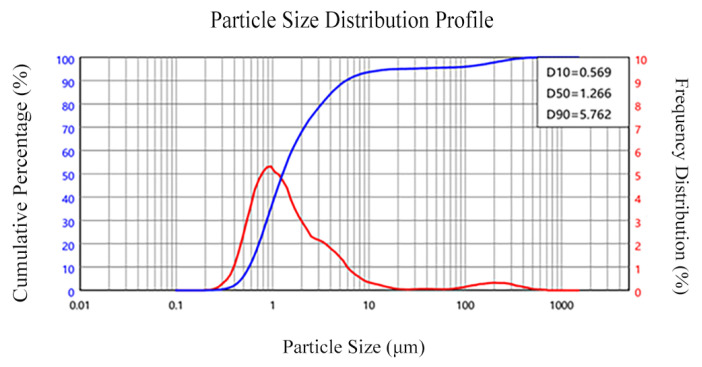
Particle size distribution of silica fume.

The following test material was acquired: OPC is ordinary Portland cement with a density of 3.03 g/cm^3^; the particle size distribution of the cement is shown in Figure 1. Fly ash was grade I fly ash, and its apparent density was 2.4 g/cm^3^; the particle size distribution of the fly ash is presented in Figure 2. The apparent density of silica fume was 2.232 g/cm^3^; the particle size distribution of silica fume is illustrated in Figure 3. And the oxide composition of the three materials is shown in Table 1. The use of fly ash and silica fume in ECC can reduce the cost of materials and increase the self-compacting and working performance of ECC when used in large quantities [17,18]. The mechanical and geometric properties of UHMWPE and BFs are shown in Table 2. Quartz sand exhibited a mean particle size distribution ranging from 0.09 to 0.12 mm, and its density was 2.654 g/cm^3^. A polycarboxylate-based high-range water reducer was employed as the chemical admixture; water was ordinary tap water.

### 2.2. Mix Proportion Design

With Design-Expert V8.0.6 software, 17 groups of three factors and three levels were designed based on previous research results and a single-factor test [19]. Each group had three replicates, three factors of which were A the water-to-binder ratio, B the content of UHMWPE fiber, and C the content of BF. The coded values for each factor are presented in Table 3, and Table 4 shows the ratio design scheme based on the Box–Behnken design.

### 2.3. Preparation and Test Methods of Samples

The uniform dispersion of fibers is a prerequisite for the full use of fibers in ECC [20]. The mixing procedure adopted in this study was as follows: add some mixing water, charge the mixer with cementitious materials and admixtures, stir at low speed for 2 min, add UHMWPE and BF and stir at high speed for 2 min, and then add the reserved mixing water, followed by high-speed mixing for 2 min. After this stage, the prepared composite material was cast in the mold, placed on the shaking table for 3 min, demolded after 24 h, and cured at room temperature (25 ± 2 °C) at about 95% relative humidity for 28 days. All mechanical property assessments were conducted on specimens following a 28-day standard curing period.

**Figure 4:**
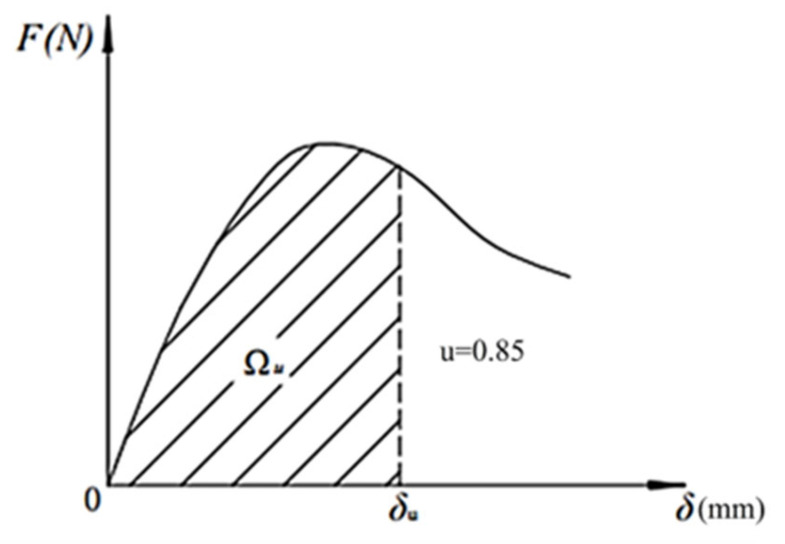
Schematic for calculating the equivalent flexural strength.

**Figure 5:**
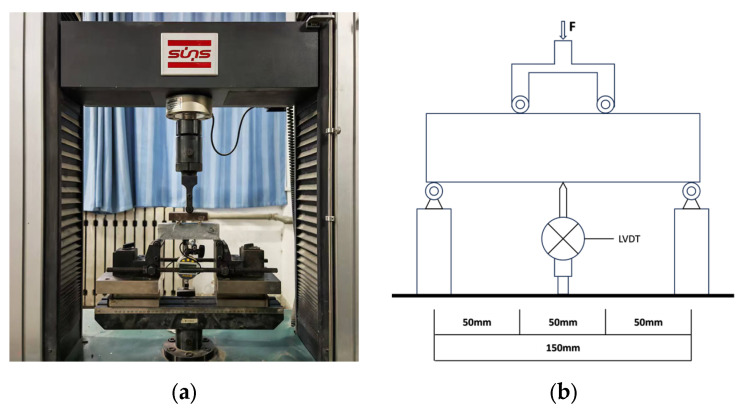
Four-point bending test setup: (**a**) four-point bending test loading method; (**b**) geometric dimensions.

The loading equipment used in the compressive and flexural tests was a YAW-300D cement mortar press-folding machine (Jinan Xingguang Testing Machine Manufacturing Co., Ltd., Jinan, China), and the compression area was 40 × 40 mm^2^. The testing procedure complied with Chinese industrial standard GB/T 17671-2021 (Test Method of Cement Mortar Strength (ISO Method)) [21]. The four-point bending test method was employed to evaluate bending toughness. The loading rate was 0.2 mm/min. The size of the specimen was 40 mm × 40 mm × 160 mm. The span between the two supports waws L = 150 mm, and the middle section was a pure bending section [22]. The equivalent bending strength was calculated according to Equation (1), and the calculated values were recorded with an accuracy of 0.1 MPa. The equivalent bending toughness Wu was calculated according to Equation (2), and the calculation result was accurate to 0.1 KJ/m^3^. Figure 4 illustrates the equivalent calculation principle of the specimen. The loading equipment was a UTM-5105 electronic universal testing machine, which adopted four-point bending loading, displacement control mode to load, and a loading rate of 0.2 mm/min. The experimental loading setup is illustrated in Figure 5.

(1) $$f_{u}=\frac{\Omega_{u}L}{bh^{2}\delta_{u}}.$$

(2) $$W_{u}=\frac{\Omega_{u}}{bh^{2}}.$$

In the above equations, $f_{u}$ is the equivalent bending strength (N/mm^2^); $W_{u}$ is the equivalent bending toughness (KJ/m^3^); h is the height of cross section (mm); b is the width of the section (mm); L is the span of the specimen (mm); $\delta_{u}$ is the corresponding deflection value when the load drops to *u* times the peak load, where *u* is 0.85; and $\Omega_{u}$ is the area under the load–deflection curve when the mid-span deflection is $\delta_{u}$.

## 3. Experiment Results and Analysis

### 3.1. Compressive Strength

**Figure 6:**
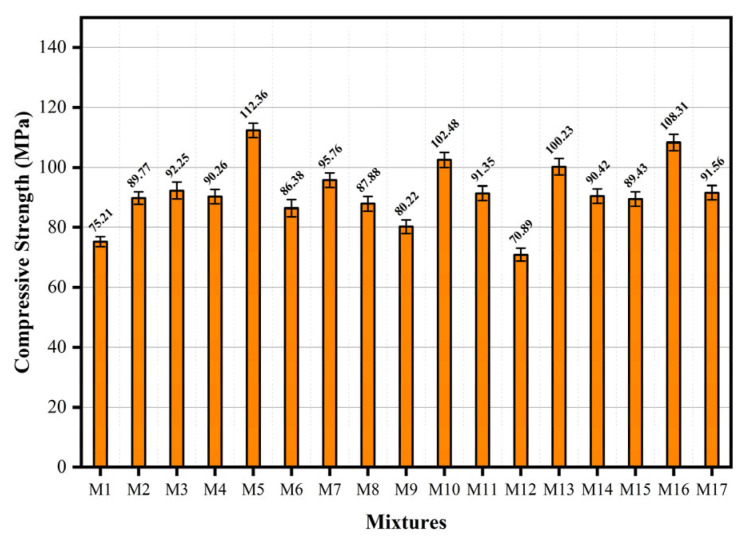
Compressive strength of hybrid fiber ECC.

The compressive strength of hybrid fiber ECC was measured at the curing age of 28 d. The compressive strength trend is illustrated in Figure 6. The maximum recorded compressive strength reached 112.36 MPa. Ratio M5 had the lowest water-to-binder ratio of 0.2 in the design. The contents of UHMWPE and BFs were 1.2% and 0.8%. At this time, the internal pores of the material were low and relatively dense. In the cases of different particle sizes, the pores formed between different particles were filled with finer particles [23]. This demonstrates superior compressive strength compared to the other components. In comparing components M6 and M17, under the same water-to-binder ratio and BF content, the UHMWPE fiber increased from 0.8% to 1.6%, and the compressive strength increased by 6.0%. The observed variations among M5, M7, M15, and M16—all with the same water-to-binder ratio—primarily arise from the synergistic effects of basalt fiber (BF) and UHMWPE fiber content. An increase in BF content refines the pore structure, thereby enhancing compressive strength, while a moderate rise in UHMWPE content improves strength through its high tensile properties. However, excessive total fiber content may reduce overall compactness and cause fiber agglomeration, leading to decreased strength. Specifically, M16 exhibits higher strength than M5 due to the increased proportion of pore-refining BFs at an optimal UHMWPE level. In contrast, M7 suffers from fiber agglomeration and porosity caused by high UHMWPE content, while M15 experiences insufficient BF activation and low UHMWPE contribution; these factors collectively contributed to the reduction in compressive performance. When the specimen was subjected to axial load, the compressive bearing surface of the specimen was slightly reduced, and a slight lateral displacement was generated. The fiber bridging effect effectively constrained the lateral displacement of the specimen, thereby sharing part of the axial pressure and improving the compressive strength of the specimen [24]. The lowest compressive strength was 70.89 MPa. Component M12 had the highest fiber content; UHMWPE and BF were both 1.6%. Relative to M14 and the reference component, under a constant water-to-binder ratio and UHMWPE content, a 21.6% reduction in compressive strength was observed with increasing BF content. When a low water-to-binder ratio was maintained, excessive fiber content was not conducive to its uniform dispersion, resulting in agglomeration in the matrix. This process effectively reduces the material’s compressive strength, as it is equivalent to introducing defects into the matrix The compressive strength model relationship was established through response surface analysis of each factor, as shown in Equation (3).

(3) $$\begin{array}{c} Y_{1}=+90.30-3.15A+2.17B-2.6C-0.29AB+0.45AC\\ \;-6.14BC+13.57A^{2}-13.09B^{2}+1.97C^{2} \end{array}$$

In the above equations, *Y*_1_ is the compressive strength; *A* is the water-to-binder ratio; *B* is the content of UHMWPE; and *C* is the content of BF.

To evaluate the accuracy of the model, an analysis of the variance of this equation was conducted. Through analysis of variance (ANOVA) at a significance level of *p* < 0.05, the significance of the second-order polynomial function was quantified. Table 5 summarizes the analysis of variance results, where PM < 0.0001 < 0.05, and the model’s statistical significance at the 95% confidence level is demonstrated. The F test results show that the F value is 36.27 (PM < 0.0001), and there is an extremely significant statistical relationship between the predictive variables and the response variables in the model. These results provide substantiating evidence for the model’s validity. When the lack-of-fit term was FL = 3.23, a *p*-value (PL) of 0.1436 was obtained, which is greater than 0.05. This indicates that the lack of fit was not significant, the model error was small, and the model demonstrated accurate fitting to the experimental data. In analyzing the *p*-value of a single factor, it can be seen that the water-to-binder ratio has the most significant effect on the compressive strength, and the content of UHMWPE and BF has a significant effect on the compressive strength.

**Figure 7:**
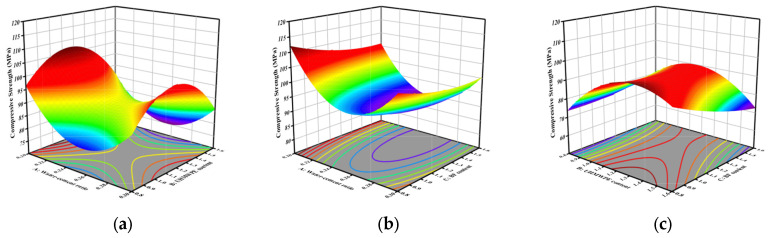
Three-dimensional response surface plot of the interaction between two factors in compressive strength: (**a**) AB interaction; (**b**) AC interaction; (**c**) BC interaction.

Compressive strength values across the parameter domain are calculable using Equation (3). Figure 7 is a 3D response surface diagram of the two-factor interaction of compressive strength. Figure 7a demonstrates the interactive effect of factors A and B on compressive strength. At fixed levels of A, the compressive strength displays a non-monotonic response to increasing B, characterized by an initial decline followed by subsequent recovery, which produces distinct curvature in the response surface. A and B play a controlling role in the compressive strength. Figure 7b shows that the response surface curvature of compressive strength is lower than that of A and C, and their interaction remains statistically insignificant. Figure 7c shows the interaction between B and C, and the response surface diagram is obviously warped. At this time, the main factor affecting the compressive strength is B.

### 3.2. Flexural Strength

**Figure 8:**
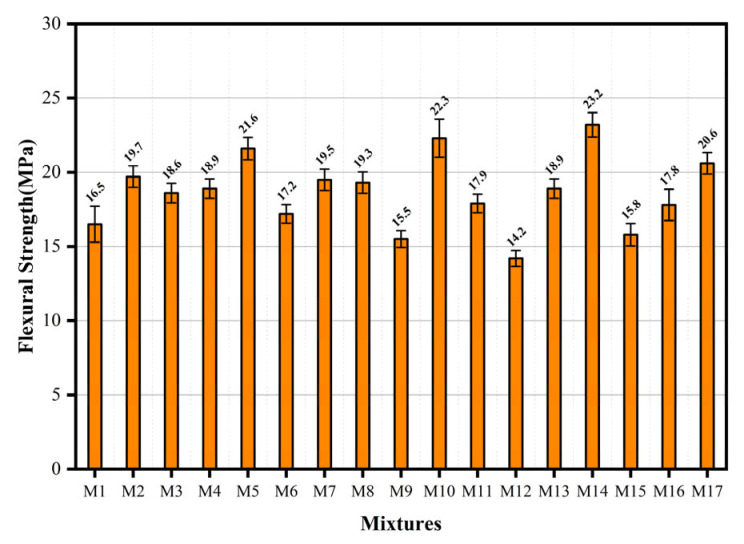
Flexural strength of hybrid fiber ECC.

Following 28 days of standard curing, an evaluation was conducted on the flexural performance of the hybrid fiber-reinforced ECC, as shown in Figure 8. The ultimate flexural strength of ECC ranged from 14.2 MPa to 23.2 MPa. To isolate the effect of PE fiber content on mechanical properties, the comparison cases were selected with the maximum water-to-binder ratio and a moderate basalt fiber content. This approach minimizes the confounding influences of matrix fluidity and basalt fiber dispersion, as the latter exhibits superior dispersion in cementitious matrices compared to organic PE fibers. By maintaining consistent water-to-binder and basalt fiber levels, variations in mechanical performance can be more reliably attributed to PE fiber dosage [25]. Compared with components M6 and M17, the flexural strength increased significantly by 19.8% when the content of UHMWPE increased from 0.8% to 1.6% under the same water-to-binder ratio and BF content. Because of the bridging effect of the fiber at the micro-crack, when the mid-span load causes the initial micro-crack of the specimen, the stress at the crack tip is lower than the fiber bridging stress, while the matrix phase suppresses crack propagation through stress redistribution mechanisms. Elevated fiber content results in higher fiber density across crack surfaces, consequently improving the bridging capacity, thereby effectively delaying crack propagation and improving the flexural strength of the specimen. Response surface analysis of the influencing factors yielded a correlation model for flexural strength, represented by Equation (4):

(4) $$\begin{array}{c} Y_{2}=+18.88+0.54A+1.56B-2.15C-0.075AB+0.1AC\\ \;-2BC+1.1A^{2}-1.7B^{2}+0.17C^{2} \end{array}$$

In the above equation, *Y*_2_ is flexural strength; *A* is the water-to-binder ratio; *B* is the content of UHMWPE; and *C* is the content of BF.

Table 6 summarizes the analysis of variance results of the model, where PM < 0.0003 < 0.05, FM = 21.62, indicating that the model can provide an excellent and accurate response. When the lack-of-fit term was FL = 0.99, a *p*-value (PL) of 0.4822 was obtained, which is greater than 0.05. This indicates that the lack of fit was not significant, the model error was small, and the model demonstrates excellent agreement with the experimental data. Regarding this sensitivity to a single factor, UHMWPE and BF content had a significant response to flexural strength, and the water-to-binder ratio exhibited statistical significance.

**Figure 9:**
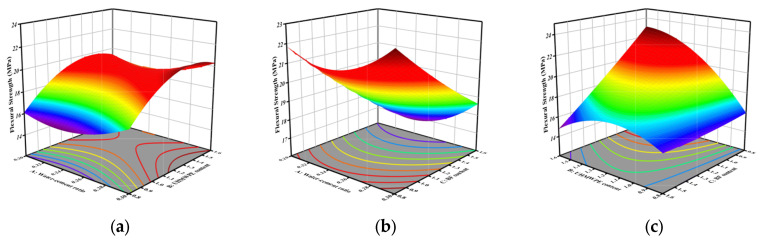
Three-dimensional response surface plot of the interaction between two factors in flexural strength: (**a**) AB interaction; (**b**) AC interaction; (**c**) BC interaction.

The flexural strength for all parameter combinations within the defined bounds is calculable using Equation (4). Figure 9 shows a 3D response surface diagram of the two-factor interaction of flexural strength. From Figure 9a, it can be seen that in the process of B increasing from 0.8% to 1.6%, the flexural strength generally shows an upward trend. Figure 9b shows the effect of AC interaction on flexural strength. At constant A levels, flexural strength exhibits a negative correlation with increasing C. Figure 9c shows the interaction of BC. The UHMWPE fiber content exhibits a statistically significant influence on flexural strength. With the increase in B, a statistically significant increasing trend is observed in flexural strength, and the interaction between UHMWPE and BF is extremely significant. The statistical test results of the *p*-value, obtained through model regression analysis, are consistent with this conclusion.

### 3.3. Equivalent Bending Toughness

**Figure 10:**
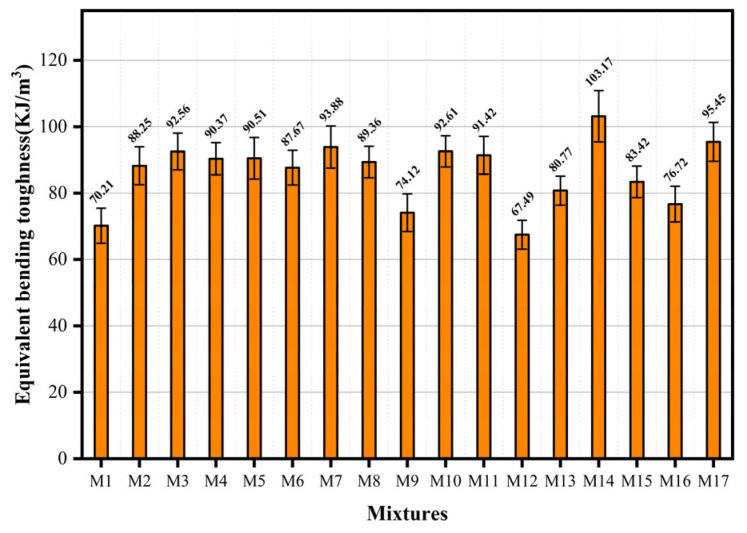
Equivalent bending toughness of hybrid fiber ECC.

At the curing age of 28 days, measurements were taken for the equivalent bending toughness of the ECC, and the variation range was 67.49–103.17 KJ/m^3^, as shown in Figure 10. The M14 mix proportion demonstrates optimal energy absorption characteristics throughout the loading process. The ratio has an intermediate level of the water-to-binder ratio and the highest UHMWPE content in the design, which were 0.25 and 1.6%, respectively. Compared with other components, the equivalent bending toughness was reduced when the material components were increased or reduced. Compared with component M1, the content of UHMWPE fiber decreased by 0.8%, and the equivalent bending toughness decreased by 32.0% under the same water-to-binder ratio. Compared with M12, the content of BF increased by 0.8%, and the flexural toughness decreased by 34.6%. The equivalent bending toughness was affected by the fiber, bulk matrix behavior, and fiber–matrix interfacial zone properties. When specimens were loaded in the four-point bending configuration, the synergistic effect between the matrix and the fiber realized high energy absorption during the loading process. With the regulation of fiber dispersion and stress tip distribution, water-to-binder ratios and fiber contents that are either too high or too low alter the toughness of the matrix. Furthermore, with an optimized water-to-binder ratio and fiber dosage, the fiber continues to increase more than the optimal content, which will cause fiber agglomeration and deterioration of distribution uniformity [26]. At the same time, excessive fibers will increase the porosity of the matrix, destroy the dense structure of the matrix, and ultimately lead to a decrease in the mechanical properties of the specimen. Finding the optimal water-to-binder ratio and fiber content is crucial in the preparation of hybrid fiber ECC specimens, as it enables the achievement of a balanced state for the material’s flexural toughness. Through the application of Response Surface Methodology to each variable, the bending toughness model relationship is established, as shown in Equation (5).

(5) $$\begin{array}{c} Y_{3}=+90.39+1.5A+5.57B-7.18C-0.67AB+0.49AC\\ \;-9.9BC+3.06A^{2}-3.35B^{2}-8.3C^{2} \end{array}$$

In the above equation, *Y*_3_ is the equivalent bending toughness; *A* is the water-to-binder ratio; *B* is the content of UHMWPE; and *C* is the content of BF.

The results of further analysis of variance for this equation are shown in Table 7. The model is significant at the 95% confidence level (CL), as indicated by PM < 0.0001 < 0.05. With the probability of F = 46.30 being less than 0.01%, the model exhibits high response sensitivity. A *p*-value (PL) of 0.1117 was obtained when the lack-of-fit term was FL = 1.51, which is greater than 0.05. This demonstrates that the lack of fit was not significant, the model error was small, and the experimental results were accurately fitted by the model.

In addition, the content of UHMWPE and BF has the highest sensitivity to flexural toughness, with low sensitivity observed for the water-to-binder ratio. Regarding factor interactions affecting flexural toughness, the UHMWPE-BF content interaction is highly significant, whereas the water-to-binder ratio–UHMWPE interaction shows no statistical significance.

**Figure 11:**
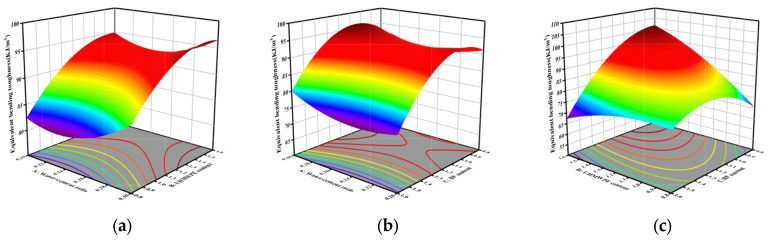
Three-dimensional response surface plot of the interaction between two factors in equivalent bending toughness strength: (**a**) AB interaction; (**b**) AC interaction; (**c**) BC interaction.

For the parameters considered within the definition range, Equation (5) can be used to determine the equivalent bending toughness. Figure 11 presents the three-dimensional response surface plot for the two-factor interaction effect on the equivalent bending toughness. Figure 11a shows that the sensitivity of the water-to-binder ratio to flexural toughness is weak, which is consistent with the conclusion that the interaction term *p*-value is large (not significant) in the above analysis. Under constant water-to-binder ratio conditions, flexural toughness exhibits a positive correlation with UHMWPE content. Figure 11b illustrates the significant interactive effect between factors A and C on flexural toughness. At any fixed level of A, the flexural toughness shows a distinct rise-and-fall pattern as C increases, confirming a statistically significant interaction between the two variables. Figure 11c shows that when B is 1.6% and C is 0.8%, it possesses optimal equivalent bending toughness performance, and the response surface graph is obviously warped, suggesting a statistically significant interaction between the two factors. The statistical test results of the *p*-value, obtained through the model regression analysis, align with this conclusion.

### 3.4. Model Feasibility Test

**Figure 12:**
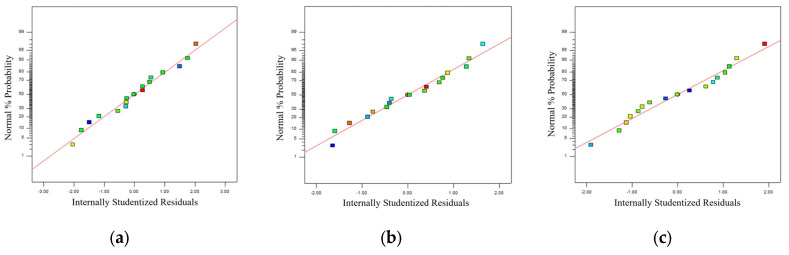
Normal plot of residuals: (**a**) compressive strength; (**b**) flexural strength; (**c**) equivalent bending toughness.

**Figure 13:**
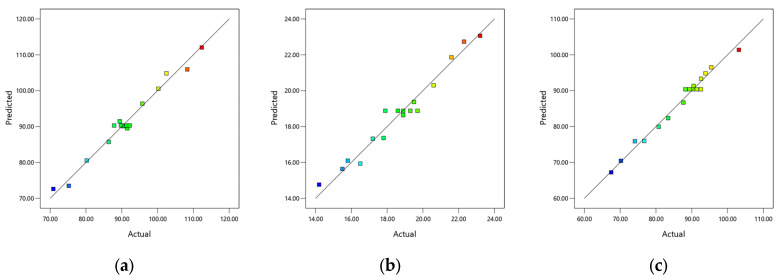
Comparison chart of experimental values and predicted values: (**a**) compressive strength; (**b**) flexural strength; (**c**) equivalent bending toughness.

The correlation coefficients R2 between experimental and predicted values are 0.9790, 0.9653, and 0.9835, respectively, as presented in Table 8 for model reliability assessment, demonstrating their close approximation to the ideal value of 1. The difference between the R^2^ and adjusted R^2^ values for each response is less than 0.2. The model achieved a coefficient of variation below 10 and a signal-to-noise ratio above 4. This shows that the reliability and accuracy of the regression equation are high, which can meet the fitting of the actual situation of the test. The residual distribution is shown in Figure 12, which is used to evaluate the normality hypothesis of the residual distribution. The significant relationship within the regression model equation involving the water-to-binder ratio and fiber content is confirmed by the even distribution of the scatter points in the graph. Figure 13 shows that the close agreement between experimental and predicted values, along with their linear distribution, demonstrates the high reliability of the regression model in predicting response values and its capability to accurately forecast experimental outcomes.

## 4. Multi-Objective Optimization and Verification of Mix Proportion

### 4.1. NSGA-II Algorithm and TOPSIS Evaluation Principle

NSGA-II is an efficient algorithm based on the concept of Pareto optimality for solving multi-objective optimization problems (MOPs), and ir introduce a fast non-dominated sorting mechanism to stratify the individuals of the population. At the same time, the crowding distance and its comparison operator are used to maintain the distribution diversity of the solution set on the Pareto front, and an elite retention strategy is implemented to preserve high-fitness individuals across generations. Its core process includes population initialization. The algorithm incorporates several key steps: non-dominated sorting, crowding distance calculation, genetic operations (selection, crossover, mutation), the merging of parent and child populations, and the screening of new generation populations. This process is iterated until the termination conditions are met. The use of a multi-criteria decision-making method is typically required to select the final solution from the obtained non-dominated solution set. TOPSIS is a classical multi-criteria decision-making method. Its core concept involves constructing positive and negative ideal solutions and fully utilizing original data information to quantitatively assess each alternative’s proximity to the ideal solution and alienation from the negative-ideal solution. The relative closeness is then computed to integrally represent these two aspects, resulting in highly objective and rational evaluation outcomes. It has been extensively applied in various fields, including vehicle engineering optimization [27], crop allocation optimization [28], electro-thermal energy conversion system optimization [29], and multi-objective optimization of residential buildings [30].

### 4.2. Objective Function and Optimization Process

**Figure 14:**
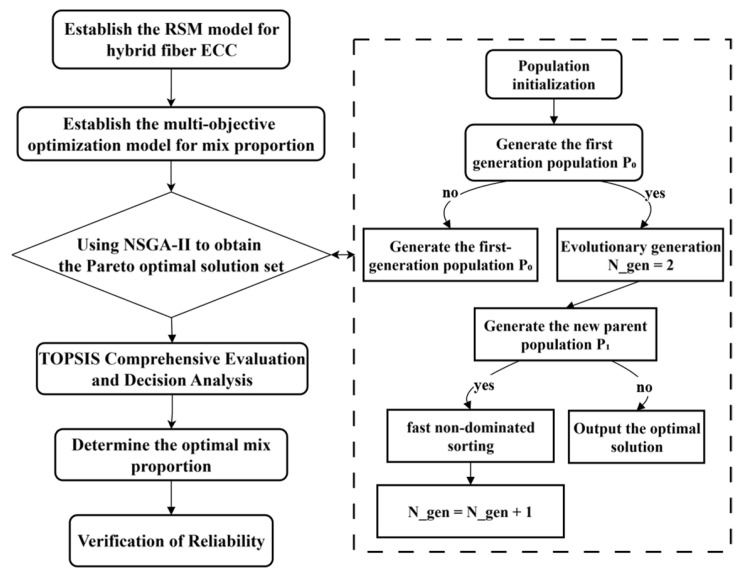
Flowchart of multi-objective optimization.

Based on the regression equation obtained by the response surface model, the NSGA-II algorithm is used to optimize the strength and toughness index parameters of the hybrid ECC for this multi-factor and multi-evaluation index optimization problem. The optimization process is shown in Figure 14, with the dual objectives of maximizing both compressive strength and equivalent bending toughness. The multi-objective optimization model, formulated in Equation (6), is established based on the preceding analysis.

(6) $$\left\{\begin{array}{c} (A^{*},B^{*},C^{*})=\mathrm{argmin}\left[-Y_{1},-Y_{3}\right]\\ \;s.t.\;A^{*}\in (0.2,\;0.3)\\ \;\;\;\;\;\;B^{*}\in (0.8\%,1.6\%)\\ \;\;\;\;\;\;C^{*}\in (0.8\%,1.6\%) \end{array}\right.$$

In the above equations, Y_1_ is the compressive strength model and Y_3_ is the equivalent bending toughness model, which correspond to the above regression equations, respectively; A*, B*, and C* represent the water-to-binder ratio, UHMWPE, and BF content, respectively; and each value range is used as the constraint condition of the optimization problem.

### 4.3. Multi-Objective Optimization and Verification

**Figure 15:**
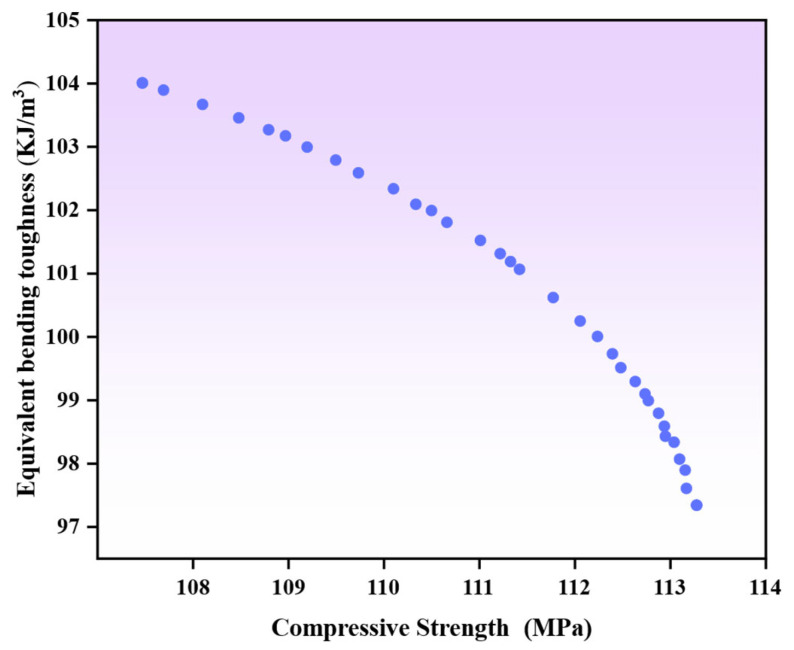
Multi-objective Pareto optimal solution set.

The established framework, which comprises prediction models for the compressive strength and equivalent bending toughness of hybrid fiber-reinforced high-ductility cementitious composites, serves as the basis for the subsequent analysis. The NSGA-II algorithm is employed for multi-objective optimization. The parameter settings are specified as follows: with crossover and mutation probabilities set at 0.9 and 0.1, respectively, the algorithm configuration also includes a population size of 50 and a maximum of 200 iterations. The Pareto front solution set obtained by the optimization calculation is shown in Figure 15. The solution set quantitatively characterizes the internal constraint relationship between the two key performance indicators of compressive strength and equivalent bending toughness. The Pareto front clearly shows that there is no single design scheme that can maximize compressive strength and equivalent bending toughness at the same time. The fact that no single solution can simultaneously optimize both objectives to their respective ideal values indicates a non-negligible trade-off relationship between the two performance metrics.

**Figure 16:**
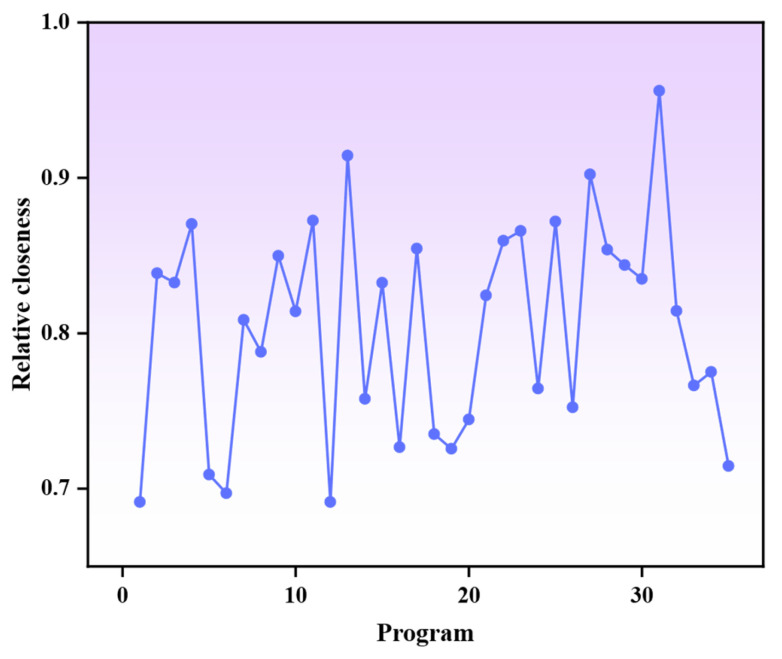
Relative closeness of the schemes.

From the Pareto optimal solution set, 35 representative solutions were evaluated using the TOPSIS method to identify the hybrid fiber ECC ratio with the best comprehensive performance. This method computes the Euclidean distance between each solution and the positive/negative ideal solutions, deriving a relative closeness value (0–1, where values closer to 1 indicate better performance) to comprehensively assess both compressive strength and equivalent bending toughness. As shown in Figure 16, the highest closeness of the 31st group scheme is 0.956, and the corresponding mix ratio as follows: a water-to-binder ratio of 0.21, UHMWPE fiber content of 1.51%, and BF content of 0.85%. This indicates that the group achieves optimal balance between strength and toughness.

Three groups of parallel tests were conducted using the model-optimized parameters, with the mean value serving as the final verification result to validate the reliability of the optimization outcomes. A comparison between predicted and experimental values is presented in Table 9, where D denotes the absolute value of the relative deviation calculated using Equation (7). The relative errors for responses Y_1_ and Y_3_ between predicted and experimental values are 3.1% and 2.9%, respectively, both below the 5% threshold. These results confirm the high significance of the established hybrid fiber ECC prediction model, verify the effectiveness of the combined NSGA-II and TOPSIS optimization strategy, demonstrate the accurate predictive capability of the regression models for compressive strength and equivalent bending toughness, and establish a foundation for a high-performance design of hybrid fiber ECC.

To validate the reliability of the optimization outcomes, three sets of parallel tests were carried out according to the optimal parameter configuration obtained by the model. Table 9 summarizes the comparative analysis of model-predicted values versus experimentally obtained data. The relative error (D) is used as the evaluation index, and the calculation method is shown in Equation (7). The accuracy and applicability of the constructed hybrid fiber ECC performance prediction model are verified by the good agreement between model predictions and actual test data, as evidenced by the relative errors of compressive strength and equivalent bending toughness being 3.1% and 2.9%, respectively—both within the 5% threshold as shown in the table. This also further confirms the effectiveness of the NSGA-II and TOPSIS joint optimization strategy in composite performance trade-off and optimal mix ratio screening, which can provide reliable theoretical support and experimental basis for the high-performance mix ratio design of hybrid fiber ECC materials.

(7) $$D=\frac{\left|Y_{T}-Y_{P}\right|}{Y_{T}}\times 100\%$$

In the above equation, *Y_T_* is the measured value, and *Y_P_* is the predicted value.

## 5. Conclusions

(1) In this investigation, Engineered Cementitious Composites were produced by incorporating ultra-high-molecular-weight polyethylene and basalt fibers. The addition of UHMWPE fibers contributes to enhanced energy absorption capacity within the cementitious matrix while improving deformation capability under stress. Experimental results demonstrate that increasing the UHMWPE fiber content from 0.8% to 1.6% yields a substantial 19.8% enhancement in flexural strength performance. The complementary interaction between these two distinct fiber types generates beneficial effects on the overall mechanical behavior of the hybrid system. It should be noted that under the conditions of a low water-to-binder ratio, total fiber incorporation exceeding 2.0% induces fiber clustering phenomena, subsequently diminishing the composite’s mechanical properties.

(2) Analysis of the Box–Behnken designed model within Response Surface Methodology reveals that fiber content exhibits substantial sensitivity toward both flexural strength and equivalent bending toughness, with a significance level surpassing 95%. The order of factor significance is established as UHMWPE content exceeding BF content, which in turn outweighs the water-to-binder ratio. Regarding two-factor interactions, the direction of correlation—whether positive or negative—is determined by the magnitude of the interaction term. The model equation was validated through analysis of variance, demonstrating a high goodness-of-fit in the results.

(3) The integration of the NSGA-II algorithm with the TOPSIS comprehensive evaluation method enabled the multi-objective optimization of compressive strength and equivalent bending toughness. The derived optimal mixture proportions consist of a water-to-binder ratio of 0.21, UHMWPE fiber content of 1.51%, and basalt fiber content of 0.85%. With relative errors between predicted and experimental values remaining below 5%, this study demonstrates the considerable advantages of Response Surface Methodology and NSGA-II in balancing strength versus toughness in hybrid fiber ECC. Furthermore, it provides valuable insights for designing cementitious composites with customizable strength–toughness characteristics.

(4) For subsequent investigations, establishing a multi-objective optimization framework that integrates hybrid fiber systems with key durability indicators—such as freeze–thaw and chemical corrosion resistance—will be pivotal to enhancing the service life and performance of ECC in harsh environments.

## Figures and Tables

**Table 1:** Chemical compositions (wt.%) of cementitious materials.

Chemical Oxide	OPC (%)	Fly Ash (%)	Silica Ash (%)
SiO_2_	16.450	65.7	93.7
Al_2_O_3_	4.788	26.1	1.3
Fe_2_O_3_	3.528	-	0.9
CaO	64.745	2.9	0.4
MgO	2.863	-	0.8
Na_2_O	0.20	0.4	1.3
SO_3_	3.695	-	-
K_2_O	-	1.3	-
MgO	-	1.2	-
Loss on ignition	1.40	2.4	1.6

**Table 2:** Physical and mechanical properties of BF and PE fibers.

Type	Length of Fiber (mm)	Diameter of Fiber (μm)	Density (g/cm^3^)	Modulus of Elasticity (GPa)	Tensile Strength (GPa)
UHMWPE fiber	12	25	0.93	122	≥3.1
BF	12	19	2.65	85	>2.2

**Table 3:** Levels and values of experimental factors.

Factor	Number	Level		
		−1	0	1
Water-to-binder Ratio	A	0.2	0.25	0.3
UHMWPE Content (%)	B	0.8	0.12	1.6
BF Content (%)	C	0.8	0.12	1.6

**Table 4:** Mix design based on Box–Behnken design.

Mixture ID	M1	M2	M3	M4	M5	M6	M7	M8	M9	M10	M11	M12	M13	M14	M15	M16	M17
A: Water-to-Binder Ratios	0.25	0.25	0.25	0.25	0.2	0.3	0.2	0.25	0.25	0.3	0.25	0.25	0.3	0.25	0.2	0.2	0.3
B: UHMWPE Content (%)	0.8	1.2	1.2	1.2	1.2	0.8	1.6	1.2	0.8	1.2	1.2	1.6	1.2	1.6	0.8	1.2	1.6
C: BF Content (%)	0.8	1.2	1.2	1.2	0.8	1.2	1.2	1.2	1.6	0.8	1.2	1.6	1.6	0.8	1.2	1.6	1.2

**Table 5:** Analysis of variance of compressive strength model.

Source	Model	A	B	C	AB	AC	BC	Lack of Fit
F	36.27	14.72	7.00	10.04	0.061	0.15	27.89	3.23
*p*-value	<0.1‰	0.0064	0.0331	0.0157	0.8116	0.7100	0.0011	0.1436

**Table 6:** Analysis of variance of flexural strength model.

Source	Model	A	B	C	AB	AC	BC	Lack of Fit
F	21.62	4.92	41.55	78.67	0.048	0.085	34.04	0.99
*p*-value	<0.3‰	0.0621	0.4%	<0.1‰	0.8331	0.779	0.0006	0.4822

**Table 7:** Analysis of variance of equivalent bending toughness.

Source	Model	A	B	C	AB	AC	BC	Lack of Fit
F	46.30	5.16	71.48	118.55	0.52	0.27	112.79	1.51
*p*-value	<0.1‰	0.0574	<0.1‰	<0.1‰	0.4955	0.6170	<0.1‰	0.3411

**Table 8:** Model credibility test analysis table.

Regression Model	R^2^	Adjusted R^2^	Predicted R^2^	Adeq Precision	C.V./%
Y_1_	0.9790	0.9520	0.7527	22.125	2.54
Y_2_	0.9653	0.9206	0.7320	15.784	3.67
Y_3_	0.9835	0.9622	0.8476	23.886	2.16

**Table 9:** Predicted and actual values after optimizing the mix ratio.

Indicator	Compressive Strength (MPa)			Equivalent Bending Toughness (MPa)		
Type	Predicted	Actual	D (%)	Predicted	Actual	D (%)
Value	110.54	107.11	3.1	101.92	104.88	2.9

## Data Availability

The original contributions presented in this study are included in the article. Further inquiries can be directed to the corresponding author.
